# Circ-RNF121 regulates tumor progression and glucose metabolism by miR-1224-5p/FOXM1 axis in colorectal cancer

**DOI:** 10.1186/s12935-021-02290-3

**Published:** 2021-11-06

**Authors:** Zhipeng Jiang, Hao Hu, Wenli Hu, Zehui Hou, Wei Liu, Zhuomin Yu, Zhiqiang Liang, Shuang Chen

**Affiliations:** 1 Department of Gastrointestinal Surgery, Laboratory of Colorectal and Pelvic Floor Diseases, Supported By National Key Clinical Discipline, Guangdong Institute of Gastroenterology, Guangdong Provincial Key, The Sixth Affiliated Hospital of Sun Yat-Sen University, No. 26 YuanCun, 2nd Heng Road, Guangzhou, 510655 Guangdong Province China; 2 Department of General Surgery, The Sixth Affiliated Hospital of Sun Yat-Sen University, Guangzhou, Guangdong Province China; 3 Department of Critical Care Medicine, The Sixth Affiliated Hospital of Sun Yat-Sen University, Guangzhou, Guangdong Province China

## Abstract

**Aim:**

Previous studies have reported that circular RNA (circRNA) is associated with the pathogenesis of CRC. This study was designed to reveal the mechanism of circ-ring finger protein 121 (circ-RNF121) in colorectal cancer (CRC).

**Materials and methods:**

The levels of circ-RNF121, microRNA-1224-5p (miR-1224-5p) and forkhead box M1 (FOXM1) were determined by quantitative real-time polymerase chain reaction (qRT-PCR). Protein level was detected by western blot. Cell proliferation was analyzed by 3-(4,5-Dimethylthazol-2-yl)-2,5-diphenyltetrazolium bromide (MTT) and cell colony formation assays. Flow cytometry analysis was performed to investigate cell apoptosis. Cell migration and invasion were investigated by transwell and wound-healing assays. Cell glycolysis was detected using glucose, lactate and ADP/ATP ratio assay kits. The binding relationship between miR-1224-5p and circ-RNF121 or FOXM1 was predicted by starBase online database, and identified by dual-luciferase reporter assay. The impacts of circ-RNF121 silencing on tumor formation in vivo were disclosed by in vivo tumor formation assay.

**Key findings:**

Circ-RNF121 and FOXM1 expression were dramatically upregulated, while miR-1224-5p expression was downregulated in CRC tissues or cells compared with control groups. Circ-RNF121 silencing repressed cell proliferation, migration, invasion and glycolysis but induced cell apoptosis in CRC, which were attenuated by miR-1224-5p inhibitor. Additionally, circ-RNF121 acted as a sponge of miR-1224-5p and miR-1224-5p bound to FOXM1. Circ-RNF121 silencing inhibited tumor growth in vivo. Furthermore, circ-RNF121 was secreted through being packaged into exosomes.

**Significance:**

The finding provided a novel insight into studying circRNA-mediated CRC therapy.

**Supplementary Information:**

The online version contains supplementary material available at 10.1186/s12935-021-02290-3.

## Introduction

Colorectal cancer (CRC) is a common aggressive tumor that ranks 3rd in incidence and 2nd in mortality for both sexes combined [1]. About 1.3 million people are diagnosed with CRC and more than 6,50,000 cases die every year [2]. At present, despite great achievements in CRC diagnosis, over 25% cases are diagnosed at a late stage [3]. Thus, profoundly unveiling the mechanism behind CRC progression is necessary to explore new therapeutic tactics for CRC.

Circular RNA (circRNA) is a noncoding RNA with high stability [4], and is generated from the exonic regions of coding gene via back-splicing [5]. Multiple researches have reported that circRNAs participate in regulating cancer development [6, 7]. In particular, circ-ring finger protein 121 (circ-RNF121), only named as circ_100876, regulates the development of digestive system-related cancers. For instance, Cao et al. indicated that circ_100876 knockdown inhibited cell proliferation and aggressiveness of esophageal squamous cell carcinoma [8]. Moreover, circ_100876 expression was upregulated, and contributed to cell growth and metastasis in gastric cancer [9]. As predicted through GSE126094 dataset, circ-RNF121 expression was dramatically upregulated in CRC tissues; however, whether circ-RNF121 modulated CRC development was still unknown.

Exosomes are a class of membrane vesicles sized from 40 to 100 nm, and drive from endosomal multivesicular bodies [10]. They contain proteins, coding RNAs or noncoding RNAs, and mediate the transfer of these molecules [11]. Published data have demonstrated that exosomes from cancers contribute to tumor development [12].

MicroRNA (miRNA) is a small noncoding RNA with about 20 nucleotides [13]. MiRNA mainly acts function via binding to the non-coding regions of target gene, further leading to mRNA degradation or translation repression [14]. Previous researches have reported some miRNAs like miR-145 [15], miR-150-5p [16] and miR-335-5p [17] play key parts in CRC progression. MiR-1224-5p commonly served as an anti-oncogene in cancer process. For instance, Lian et al. showed long non-coding RNA (lncRNA) linc00460 contributed to osteosarcoma cell growth by binding to miR-1224-5p [18]. MiR-1224-5p repressed cell proliferation and invasion of ovarian cancer [19] and keloid [20]. In CRC, it has been revealed that miR-1224-5p represses CRC cell processes via interacting with Sp1 transcription factor (SP1) [21]. Forkhead box M1 (FOXM1), an important transcription factor, is the member of FOX superfamily [22]. The protein is commonly overexpressed in various cancers, and is involved in cell proliferation, growth and metastasis [23, 24]. Considerable research has revealed that FOXM1 contributes to cell growth and metastasis in CRC [25]. As predicted by starBase online database (http://starbase.sysu.edu.cn/agoClipRNA.php?source), miR-1225-5p contained the binding sequence of FOXM1.

Herein, the effects of circ-RNF121 silencing on cell proliferation, metastasis, apoptosis and glycolysis were analyzed. Additionally, whether circ-RNF121 regulated FOXM1 expression through sponging miR-1224-5p was testified. Furthermore, whether circ-RNF121 was secreted by CRC cells through being packaged into exosomes was unveiled.

## Materials and methods

### Tissue collection and storage

CRC patients from the Sixth Affiliated Hospital of Sun Yat-sen University provided 29 pairs of CRC tissues, including 14 CRC cases with low circ-RNF121 expression and 15 CRC cases with high circ-RNF121 expression), and paracancerous normal colorectal tissues with signing the written informed consents. All tissues were kept at -80˚C. The Ethics Committee of the Sixth Affiliated Hospital of Sun Yat-sen University agreed with this study. The association between circ-RNF121 expression and clinicopathologic features of CRC patients was shown in Additional file 3: Table S1.

### Cell culture and treatment

Otwo Biotech (Shenzhen, China) provided normal colonic epithelial cell-line NCM460, and Procell (Wuhan, China) provided CRC cell lines (SW620, Lovo, HCT-116 and SW480). Cells were cultured in Roswell Park Memorial Institute-1640 (RPMI-1640; Procell) or Dulbecco’s modified Eagle’s medium (DMEM; Procell), with 10% fetal bovine serum (FBS; Procell) and antibiotics (100 μg/mL penicillin, 100 μg/mL streptomycin) (Procell) at 37˚C in an incubator with 5% CO_2_.

In order to explore whether circ-RNF121 was secreted by being packaged into exosomes, GW4869 (10 μM; Umibio, Shanghai, China), an exosome release inhibitor, was incubated with HCT-116 and SW480 cells for 24 h.

### Plasmid construction and cell transfection

Small interfering RNA and small hairpin RNA against circ-RNF121 (si-circ-RNF121 and sh-circ-RNF121), the mimic of miR-1224-5p (miR-1224-5p), the inhibitor of miR-1224-5p (anti-miR-1224-5p) and their controls (si-NC, sh-NC, miR-NC and anti-miR-NC) were synthesized by Ribobio Co., Ltd., (Guangzhou, China). The overexpression plasmid of circ-RNF121 (circ-RNF121) was built by inserting the complete sequence of circ-RNF121 into pCD5-ciR (vector; Geneseed, Guangzhou, China). The overexpression plasmid of FOXM1 (FOXM1) was built by inserting its coding sequence into pcDNA3.1(+) vector (pcDNA) (EK-Bioscience, Shanghai, China). Si-circ-RNF121, miR-1224-5p, anti-miR-1224-5p, FOXM1 and their controls were used to investigate whether circ-RNF121 regulated CRC cell processes through FOXM1/miR-1224-5p. Circ-RNF121 and vector were employed to determine the effect of circ-RNF121 on miR-1224-5p expression. Sh-circ-RNF121 and sh-NC were used to determine the impacts of circ-RNF121 silencing on tumor growth in vivo. The synthesized sequences were si-circ-RNF121 5′-ACGCTCCTACAATGTTGATAT-3′, miR-1224-5p 5′-GUGAGGACUCGGGAGGUGG-3′, anti-miR-1224-5p 5′-CCACCUCCCGAGUCCUCAC-3′, si-NC 5′-CCATCCAAGTCAGTTTACCGATAAA-3′, miR-NC 5′-UUUGUACUACACAAAAGUACUG-3′ and anti-miR-NC 5′-CAGUACUUUUGUGUAGUACAAA-3′.

### Quantitative real-time polymerase chain reaction (qRT-PCR)

Tissues, cells and exosomes were lysed using TRIzol (TaKaRa, Dalian, China), and RNA was isolated with an RNAsimple kit (Tiangen, Beijing, China). After that, cDNA was synthesized with a primeScript™ synthesis kit (TaKaRa) or MiX-x™ miRNA synthesis Kit (TaKaRa). For the detection of the expression levels of circRNA/miRNA/mRNA, SYBR^®^ Premix DimerEraser Kit (TaKaRa) was employed. Data were assessed by the 2^−∆∆Ct^ method with U6 and β-actin as references. The sequences of sense and antisense primers were circ-RNF121 5′-CTCATCGCAACCTTGGTG-3′ and 5′-GACCCTCCATTGCTCTTCT-3′, RNF121 5′-ACCGTGGCCATGAAGCTATG-3′and 5′-GGTCACCATATTGTAGGAGCGT-3′, miR-1224-5p 5′-ACACTCCAGCTGGGGTGAGGACTCGGG-3′ and 5′-TGGTGTCGTGGAGTCG-3′, FOXM1 5′-CTTCTGGACCATTCACCC-3′ and 5′-CTCTGGATTCGGTCGTTT-3′, U6 5′-CTCGCTTCGGCAGCACA-3′ and 5′-AACGCTTCACGAATTTGCGT-3′, β-actin 5′-CACCATTGGCAATGAGCGGTTC-3′ and 5′-AGGTCTTTGCGGATGTCCACGT-3′.

### RNase R resistance analysis of circ-RNF121

RNA from HCT-116 and SW480 cells was isolated and incubated with RNase R (Geneseed) or without at a concentration of 3 U/μg RNA at 37 °C for 30 min. After that, Qiagen RNeasy MinElute Kit (Valencia, CA, USA) was chosen to purify RNA. Following that, cDNA was synthesized and circ-RNF121 amount was detected by qRT-PCR. Linear RNF121 was employed as a control.

### 3-(4,5)-dimethylthiahiazo (-z-y1)-3,5-di-phenytetrazoliumromide (MTT) assay

HCT-116 and SW480 cells were seeded in 96-well plates (5000 cells per well) for 18 h. Cells were cultured for another 48 h after various treatments. Then, MTT solution (Beyotime, Shanghai, China) was used to incubate cells. Three hours later, dimethyl sulfoxide (Sigma, St. Louis, MO, USA) was used to dissolve formazan. Cell viability was determined via detecting the output at OD 570 nm with a microplate reader (BioTek, Winooski, VT, USA).

### Cell colony formation assay

HCT-116 and SW480 cells were grown in 6-well plates (500 cells per well) for 2 weeks. RPMI-1640 medium (Procell) was replaced every 3 days during culture. Proliferative colonies were immobilized with paraformaldehyde (Sigma) and dyed with crystal violet (Sigma). Cell colony-forming ability was determined by calculating the number of colonies (containing over 50 cells).

### Flow cytometry analysis

The apoptosis of CRC cells was detected by an Annexin V-fluorescein isothiocyanate (Annexin V-FITC) apoptosis detection kit (Solarbio, Beijing, China). In short, cells were washed with phosphate buffer solution (PBS; Solarbio) and then suspended in binding buffer (Solarbio). After that, the cells were collected by centrifuging, and incubated with Annexin V-FITC (Solarbio) and propidium iodide (PI; Solarbio) in the dark, respectively. Samples were assessed using flow cytometry (Thermo Fisher, Waltham, MA, USA).

### Transwell migration and invasion assays

The migration and invasion of CRC cells were analyzed with transwell chambers without or with Matrigel (100 μL; Corning, Madison, New York, USA). In brief, HCT-116 and SW480 cells were mixed with serum-free RPMI-1640 medium (Procell) and then added into the upper chamber at a density of 1 × 10^5^ cells per well. Meanwhile, RPMI-1640 medium supplemented with 15% FBS (Procell) were added into the lower chambers. At 24 h after culture, methanol (Sigma) and crystal violet (Sigma) were used to immobilize and stain cells, respectively, in the lower chamber. Results were assessed via determining the number of the cells from six random fields under microscope (Nikon, Tokyo, Japan) with 100 ×  magnification.

### Wound-healing assay

The migratory ability of CRC cells was analyzed by wound-healing assay. Shortly, cells were cultivated in 6-well plates (2 × 10^5^ cells per well), and wounds were created when the confluence of cells reached  ~ 90%. Twenty-four hours later, results were analyzed under microscope (Nikon) with 40 ×  magnification.

### The assessment of glucose consumption and lactate production

Glucose consumption and lactate production were determined by glucose assay kit and lactate assay kit, respectively. In short, cells were collected, and then fully suspended in assay buffer (Abcam, Cambridge, UK). Then, the supernatant was collected by centrifuging. Samples were purified using Deproteinizing Sample Preparation Kit (Abcam). Following that, assay buffer (Abcam), probe (Abcam) and enzyme mix (Abcam) were added, and glucose uptake and lactate production were assessed with a microplate reader (BioTek).

### The determination of ATP/ADP ratio

The value of ATP/ADP was determined using ADP/ATP ratio assay kit (Abcam). Briefly, HCT-116 and SW480 cells were harvested after various treatments. Nucleotide Releasing Buffer (Abcam) was used to incubate the cells about 5 min. ATP Monitoring Enzyme (Abcam) diluted in Nucleotide Releasing Buffer (Abcam) was added into the test plates. At last, samples were analyzed by luminometer (Promega, Madison, WI, USA). In order to detect ADP levels, ADP-Converting enzyme (Abcam) was placed into the test wells, and samples were detected using luminometer (Promega).

### Dual-luciferase reporter assay

The binding sites between miR-1224-5p and circ-RNF121 or FOXM1 were predicted by starBase online database (http://starbase.sysu.edu.cn/agoClipRNA.php?source). The wile-type (WT) plasmids of circ-RNF121 (WT-circ-RNF121) and the 3′-untranslated region of FOXM1 (WT-FOXM1 3′UTR) were built by inserting the sequences of circ-RNF121 and FOXM1 3′UTR possessing the binding sites of miR-1224-5p into pmirGLO (Promega). The mutant (MUT) plasmids of circ-RNF121 (MUT-circ-RNF121) and FOXM1 3′UTR (MUT-FOXM1 3′UTR) were built by Geneseed Co., Ltd. The reporter plasmids were transfected into HCT-116 and SW480 cells with miR-1224-5p or miR-NC using Lipofectamine 2000 (Thermo Fisher). Dual-Lucy Assay Kit (Solarbio) was employed to detect luciferase activities with Renilla luciferase activity as a control.

### Western blot analysis

Tissues, cells or exosomes were firstly lysed using RIPA buffer (Beyotime) and then mixed with loading buffer (Thermo Fisher). The lysates were loaded onto 12% bis–tris-acrylamide gels (Thermo Fisher). Protein bands were electrotransferred onto polyvinylidene fluoride membranes (Sigma). Primary antibodies and horseradish peroxidase-labeled secondary antibody (1:2000; CST, Boston, MA, USA) were used to incubate the membranes. Finally, protein bands were visualized by RapidStep ECL Reagent (Sigma). β-actin acted as a reference. Primary antibodies were anti-FOXM1 (1:1500; Affinity, Nanjing, China), anti-clusters of differentiation 63 (anti-CD63) (1:1500; Affinity), anti-CD81 (1:1500; Affinity), anti-hexokinase 2 (anti-HK2; 1:1500; Affinity), anti-pyruvate kinase M2 (anti-PKM2; 1:1500; Affinity) and anti-β-actin (1:1000; CST).

### In vivo tumor formation assay

Male BALB/c nude mice were purchased from Charles River (Beijing, China). Mice were divided into 2 groups (sh-NC group and sh-circ-RNF121 group, N  =  7 per group). 3 × 10^6^ SW480 cells stably transfected with sh-NC or sh-circ-RNF121 were diluted in 200 μL PBS, which were then hypodermically injected into the right flank of back of mice. One week later, tumor volume was measured every 3 days. At the 25th day after injection, all mice were treated with xylazine (10 mg/kg; Seebio Biotech, Shanghai) and then euthanized by cervical dislocation. A part of each tumor was kept at – 80 °C for further study. The Animal Care and Use Committee of the Sixth Affiliated Hospital of Sun Yat-sen University agreed with this study.

### Exosome extraction and identification

Exosomes were extracted with an exosome isolation kit (Umibio). In brief, cell supernatant was collected, and then mixed with exosome concentration solution (Umibio). After cell supernatant was removed by centrifugation, the precipitates containing exosomes were collected. The extracted vesicles were identified with transmission electron microscopy (TEM; Thermo Fisher) and western blot.

### Statistical analysis

Data were assessed with SPSS 21.0 software (IBM, Somers, NY, USA) based on 3 replicates. Data were shown as means  ±  standard deviations (SD). Spearman correlation analysis was performed to analyze the linear relationships between miR-1224-5p and circ-RNF121 or FOXM1. Significant differences were compared with two-tailed Student’s *t* tests, Wilcoxon rank-sum test, log-rank test or one-way analysis of variance (ANOVA). *P* value  < 0.05 was considered statistically significant.

## Results

### Circ-RNF121 expression was upregulated in CRC tissues and cells with poor prognosis of CRC patients

**Fig. 1:**
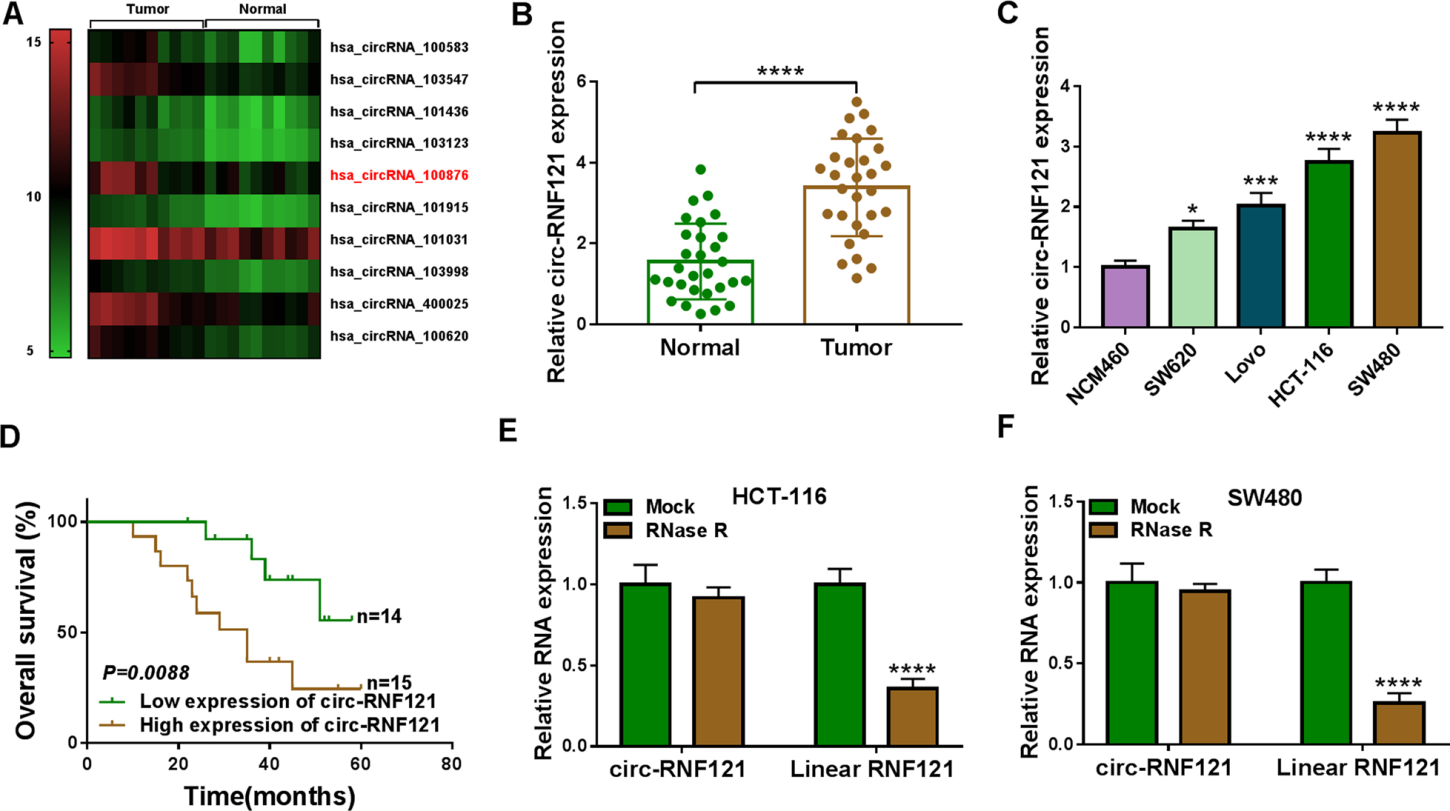
Circ-RNF121 expression was upregulated in CRC tissues and cells. **A** Heatmap showing the differently expressed circRNAs in CRC tissues in comparison normal colorectal tissues. **B**, **C** Circ-RNF121 expression was detected by qRT-PCR in 29 pairs of CRC tissues and paracancerous normal tissues as well as NCM460, SW620, Lovo, HCT-116 and SW480 cells. **D** Kaplan–Meier method was performed to assess the overall survival of CRC patients. **E**, **F** RNase R resistance analysis of circ-RNF121 assay was used to demonstrate circ-RNF121 was a circular RNA. *P  < 0.05, ***P  < 0.001 and ****P  < 0.0001

In order to screen the important circRNAs in regulating CRC cell malignancy, GSE126094 dataset was performed. Results showed that circ_100876 (circ-RNF121) expression was higher in CRC tissues than in paracancerous normal tissues (Fig. 1A). The expression level of circ-RNF121 was then detected in CRC tissues and cells. Results showed that circ-RNF121 expression was significantly upregulated in CRC tissues and SW620, Lovo, HCT-116 and SW480 cells compared with paracancerous normal tissues and NCM460 cells, respectively (Fig. 1B, C). HCT-116 and SW480 cells were selected for following studies based on higher circ-RNF121 expression in the two types of cells. Subsequently, we found that CRC patients with high circ-RNF121 expression had a poor overall survival as compared to these CRC patients with low circ-RNF121 expression (Fig. 1D). Furthermore, RNase R resistance analysis of circ-RNF121 assay displayed that circ-RNF121 expression had no apparent change after RNase R treatment, while there was a about three-fold decrease in linear RNF121 expression (Fig. 1E, F), implicating the high stability of circ-RNF121. These results suggested that circ-RNF121 might participate in CRC progression.

### Circ-RNF121 silencing repressed cell proliferation, migration, invasion and glycolysis but induced cell apoptosis in CRC

**Fig. 2:**
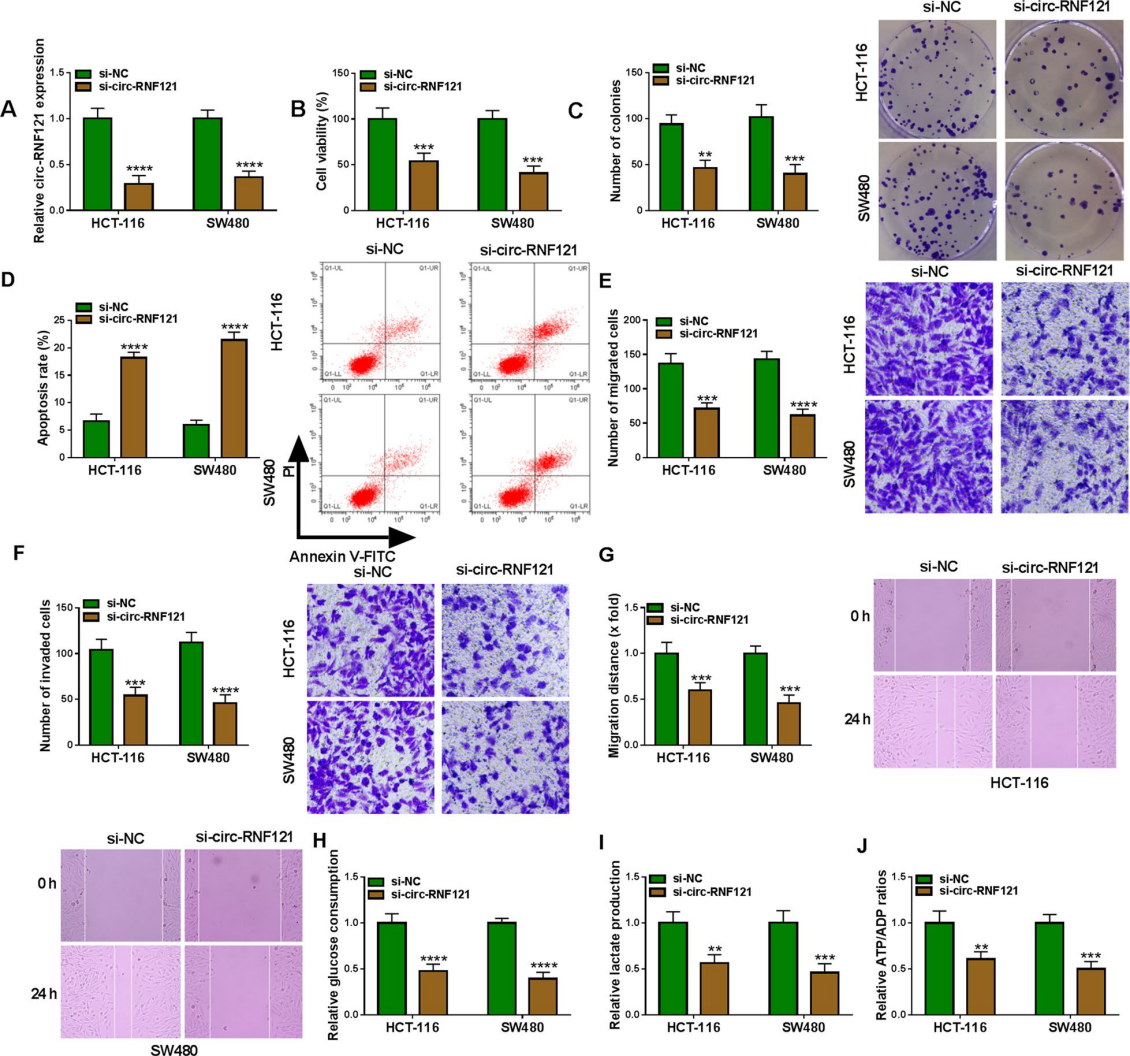
Circ-RNF121 silencing repressed CRC progression and cell glycolysis. **A** The silencing efficiency of si-circ-RNF121 was detected by qRT-PCR in HCT-116 and SW480 cells. **B**, **C** MTT assay and cell colony formation assay were performed to investigate the impact of circ-RNF121 silencing on cell proliferation in HCT-116 and SW480 cells. **D** The impact of circ-RNF121 knockdown on the apoptosis of HCT-116 and SW480 cells was determined by flow cytometry assay. **E**, **G** Transwell migration assay and wound-healing assay were carried out to analyze the influence of circ-RNF121 repression on the migration of HCT-116 and SW480 cells. **F** The effect of circ-RNF121 silencing on cell invasion was disclosed by transwell invasion assay in HCT-116 and SW480 cells. **H**, **I** Glucose assay kit and lactate assay kit were employed to determine the effects of circ-RNF121 knockdown on glucose consumption and lactate production, respectively, in HCT-116 and SW480 cells. **J** ADP/ATP ratio assay kit was performed to determine the ratio of ATP and ADP. **P  < 0.01, ***P  < 0.001 and ****P  < 0.0001

The effects of circ-RNF121 knockdown on CRC cell malignancy were further explored. The efficiency of si-circ-RNF121 in reducing circ-RNF121 expression was firstly determined. Results showed that circ-RNF121 expression was significantly downregulated in both the HCT-116 and SW480 cells transfected with si-circ-RNF121 compared with that in HCT-116 and SW480 cells transfected with si-NC (Fig. 2A). Subsequently, data showed that circ-RNF121 silencing repressed cell viability (almost 50% cells were affected) and reduced the number of cell colonies (almost 60% of total colonies) in HCT-116 and SW480 cells (Fig. 2B, C). On the contrary, circ-RNF121 silencing induced the apoptosis of HCT-116 and SW480 cells, accompanied by almost three-fold increase of cell apoptotic rate in si-circ-RNF121 group compared with si-NC group (Fig. 2D). Consistently, Fig. 2E, F showed that circ-RNF121 silencing reduced the number of migrated cells (about 52% of HCT-116 cells and 43% of SW480 cells were affects), and inhibited cell invasion (52.24% of HCT-116 cells and 41.07% of SW480 cells were affects). In support, the distance migrated by HCT-116 cells and SW480 cells were 1.67-fold and 2.17-fold shorter, respectively, in si-circ-RNF121 group than in si-NC group (Fig. 2G). Additionally, circ-RNF121 silencing suppressed glucose consumption and lactate production, and decreased ATP/ADP value. For instance, glucose consumption was 2.08-fold decreased in HCT-116 cells and 2.56-fold decreased in SW480 cells after circ-RNF121 silencing (Fig. 2H). Lactate production was about 1.70-fold decreased after circ-RNF121 silencing (Fig. 2I). ATP/ADP value was 1.67-fold lower in HCT-116 cells and 2.00-fold lower in SW480 cells after circ-RNF121 knockdown than in control groups (Fig. 2J). The above data demonstrated that circ-RNF121 silencing repressed CRC progression and cell glycolysis.

### Circ-RNF121 functioned as a sponge of miR-1224-5p

**Fig. 3:**
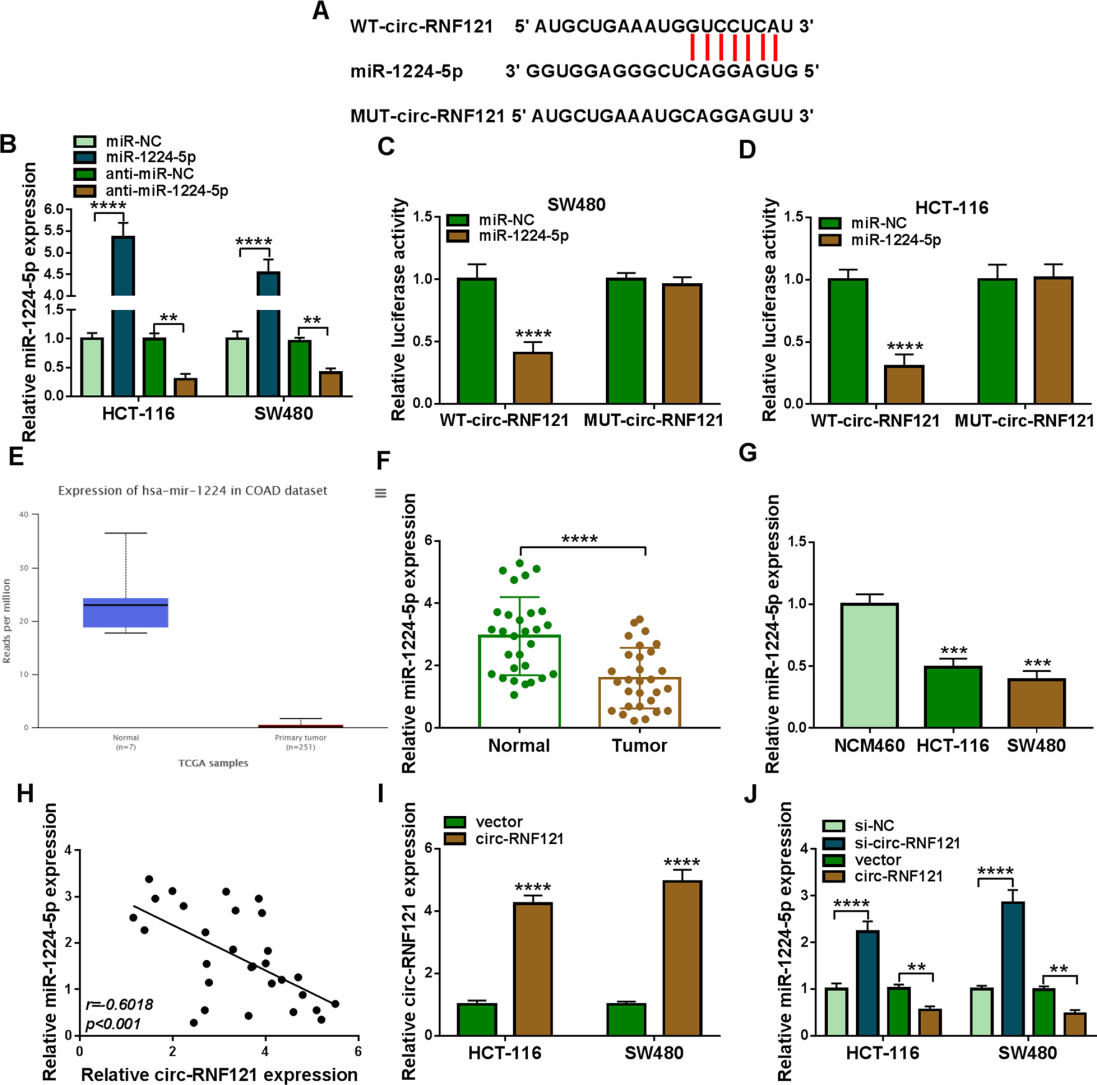
Circ-RNF121 bound to miR-1224-5p in HCT-116 and SW480 cells. **A** StarBase online database was used to predict the putative binding sites of circ-RNF121 for miR-1224-5p. **B** The efficiency of miR-1224-5p mimic and inhibitor in increasing or decreasing miR-1224-5p expression was determined by qRT-PCR in HCT-116 and SW480 cells. **C**, **D** Luciferase activities were detected by dual-luciferase reporter assay in HCT-116 and SW480 cells. **E** MiR-1224 expression was predicted by TCGA dataset in CRC tissues (N  = 251) and normal colorectal tissues (N  = 7). **F**, **G** MiR-1224-5p expression was determined by qRT-PCR in 29 pairs of CRC and paracancerous normal tissues and NCM460, HCT-116 and SW480 cells. **H** Spearman correlation analysis was performed to determine the linear relationship between circ-RNF121 and miR-1224-5p expression in CRC tissues. **I** The efficiency of circ-RNF121 overexpression was detected by qRT-PCR in HCT-116 and SW480 cells. **J** The effects of circ-RNF121 silencing and overexpression on miR-1224-5p expression were determined by qRT-PCR in HCT-116 and SW480 cells. **P  < 0.01, ***P  < 0.001 and ****P  < 0.0001

In order to reveal the mechanism of circ-RNF121 in regulating CRC cell malignancy, circ-RNF121-associated miRNA was predicted by starBase online database. The sequencing chromatograms of original and mutated sites of circ_RNF121 were presened in Additional file 1: Figrue S1. Results showed that circ-RNF121 contained the binding sites of miR-1224-5p (Fig. 3A). Subsequently, miR-1224-5p expression was significantly upregulated after transfection with miR-1224-5p mimic and downregulated after transfection with miR-1224-5p inhibitor (Fig. 3B), suggesting the success of miR-1224-5p overexpression or knockdown. Dual-luciferase reporter assay showed that luciferase activity was repressed after co-transfection with WT-circ-RNF121 and miR-1224-5p, but there was no significant change after MUT-circ-RNF121 and miR-1224-5p co-transfection (Fig. 3C, D). Additionally, miR-1224 was lowly expressed in CRC tissues in comparison with normal tissues, as predicted by TCGA dataset (Fig. 3F). Also, qRT-PCR results further showed that miR-1224-5p expression was significantly downregulated in CRC tissues and HCT-116 and SW480 cells as compared to paracancerous normal tissues and NCM460 cells, respectively (Fig. 3F, G). Spearman correlation analysis disclosed that miR-1224-5p expression was negatively related to circ-RNF121 expression in CRC tissues (Fig. 3H). Furthermore, the effects of circ-RNF121 silencing and circ-RNF121 overexpression on miR-1224-5p expression were revealed. The data from Fig. 3I showed the high efficiency of circ-RNF121 overexpression. Subsequent data displayed that miR-1224-5p expression was dramatically upregulated after circ-RNF121 silencing, and downregulated by circ-RNF121 overexpression in HCT-116 and SW480 cells (Fig. 3J). These results demonstrated that circ-RNF121 was associated with miR-1224-5p.

### Circ-RNF121 regulated tumor development and cell glycolysis by sponging miR-1224-5p in CRC

**Fig. 4:**
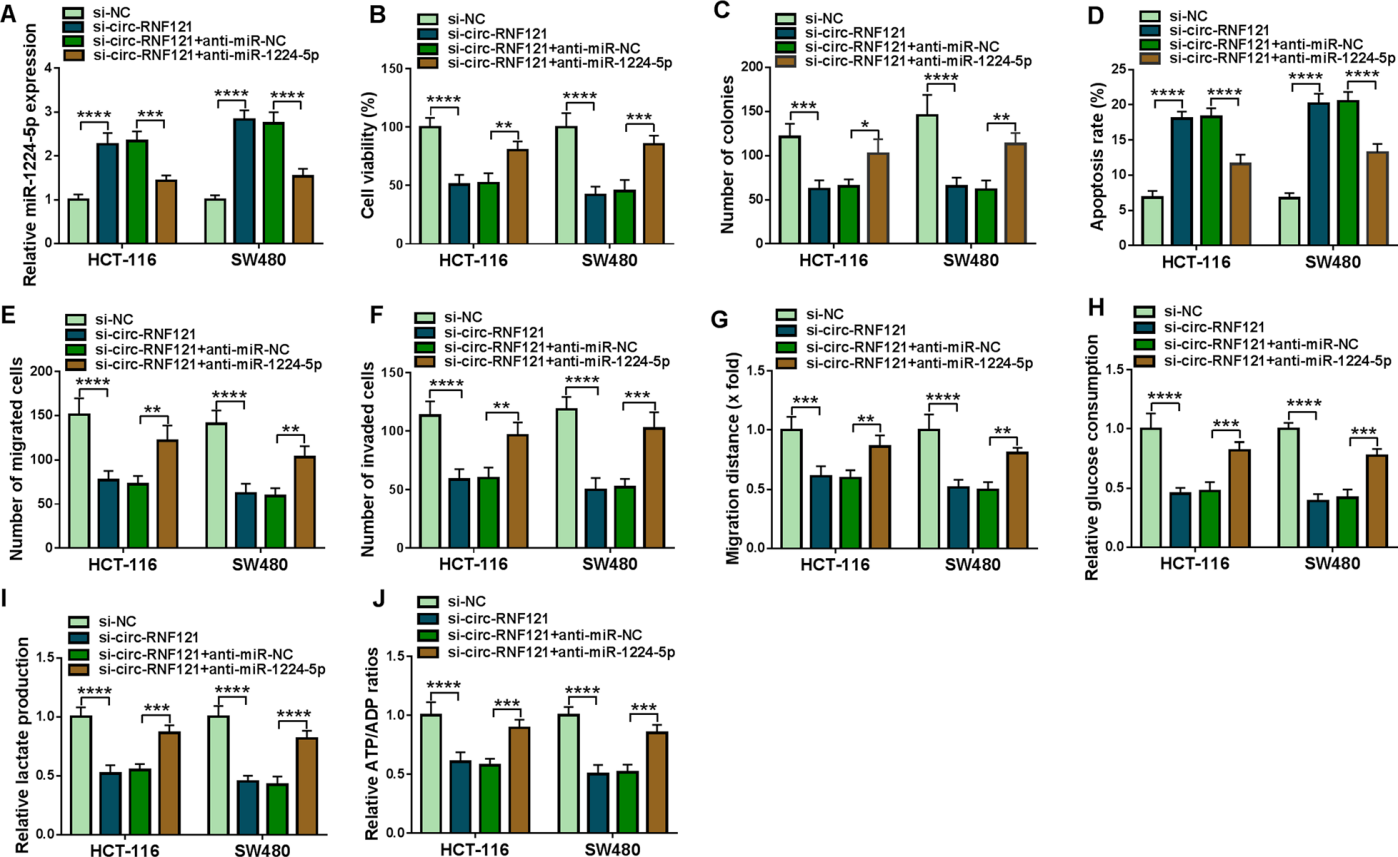
Circ-RNF121 modulated CRC cell malignancy through binding to miR-1224-5p. **A** The effects between circ-RNF121 silencing and miR-1224-5p inhibitor on miR-1224-5p expression were revealed by qRT-PCR in HCT-116 and SW480 cells. **B**, **C** The impacts between circ-RNF121 repression and miR-1224-5p inhibitor on cell proliferation were determined by MTT and cell colony formation assays in HCT-116 and SW480 cells. **D** The influences between circ-RNF121 knockdown and miR-1224-5p inhibitor on the apoptosis of HCT-116 and SW480 cells were revealed by flow cytometry analysis. **E**, **G** Transwell migration and wound-healing assays were performed to investigate the effects between circ-RNF121 repression and miR-1224-5p inhibitor on the migration of HCT-116 and SW480 cells. **F** The impacts between circ-RNF121 silencing and miR-1224-5p inhibitor on the invasion of HCT-116 and SW480 cells were demonstrated by transwell invasion assay. **H**–**J** Glucose assay kit, lactate assay kit and ADP/ATP ratio assay kit were employed to determine the effects between circ-RNF121 silencing and miR-1224-5p inhibitor on glycolysis in HCT-116 and SW480 cells. *P  < 0.05, **P  < 0.01, ***P  < 0.001 and ****P  < 0.0001

Given the binding relationship between circ-RNF121 and miR-1224-5p, whether circ-RNF121 modulated CRC cell processes via sponging miR-1224-5p was further investigated. Results firstly showed that circ-RNF121 silencing upregulated miR-1224-5p expression, whereas this effect was reversed after transfection with miR-1224-5p inhibitor (Fig. 4A). Subsequently, circ-RNF121 knockdown repressed cell viability and colony-forming ability, but miR-1224-5p inhibitor restored these impacts (Fig. 4B, C). MiR-1224-5p inhibitor also restrained the promoting effect of circ-RNF121 repression on the apoptosis of HCT-116 and SW480 cells (Fig. 4D). Additionally, the inhibitory impacts of circ-RNF121 silencing on the migration and invasion of HCT-116 and SW480 cells were partly abolished after miR-1224-5p knockdown (Fig. 4E–G). Consistently, the repressive influences of circ-RNF121 knockdown on glucose uptake and lactate production were impaired when miR-1224-5p was downregulated (Fig. 4H, I). Furthermore, circ-RNF121 knockdown reduced the value of ATP/ADP; however, miR-1224-5p inhibitor attenuated this result (Fig. 4J). Thus, the above findings manifested that circ-RNF121 could regulate cell glycolysis and CRC progression by sponging miR-1224-5p.

### MiR-1224-5p was associated with FOXM1 in CRC cells

**Fig. 5:**
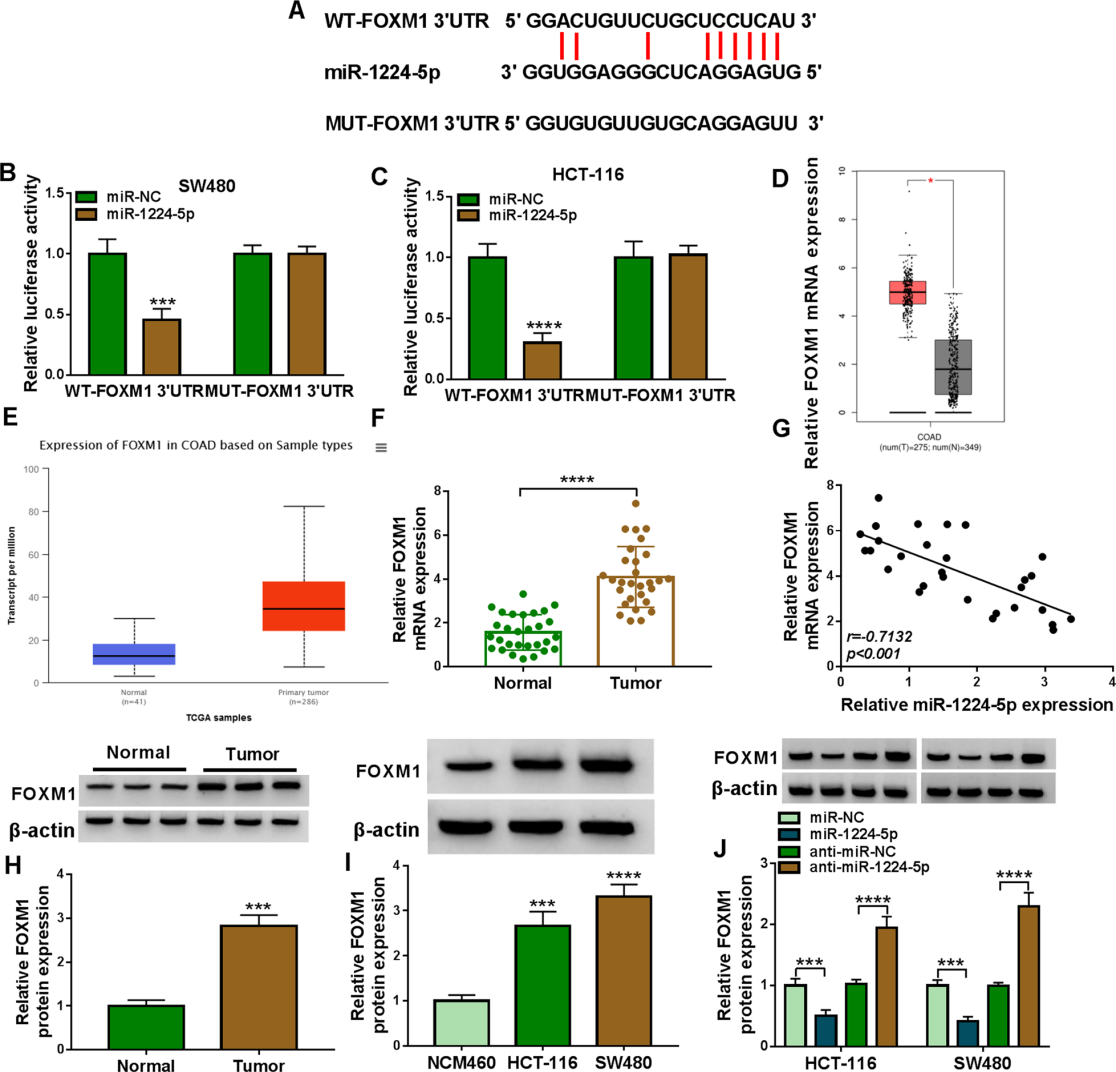
MiR-1224-5p bound to FOXM1 in CRC cells. **A** StarBase online database showed that the putative binding sites of miR-1224-5p for FOXM1 3′UTR. **B**, **C** Luciferase activity was detected by dual-luciferase reporter assay in HCT-116 and SW480 cells. **D**, **E** GEPIA and TCGA datasets were employed to assess FOXM1 expression in CRC tissues and normal colorectal tissues. **F** The mRNA level of FOXM1 was determined by qRT-PCR in 29 pairs of CRC and paracancerous normal tissues. **G** The linear relationship between miR-1224-5p and FOXM1 expression was analyzed by Spearman correlation analysis. **H**, **I** The protein level of FOXM1 was determined by western blot in 29 pairs of CRC and paracancerous normal tissues and NCM460, HCT-116 and SW480 cells. **J** The impacts of miR-1224-5p mimic and inhibitor on FOXM1 protein expression were revealed by western blot. *P  < 0.05, ***P  < 0.001 and ****P  < 0.0001

The study continued to analyze the target gene of miR-1224-5p. The sequencing chromatograms of original and mutated sites of FOXM1 3′UTR were presened in Additional file 1: Figure S1. As presented in Fig. 5A, FOXM1 3′UTR possessed the binding sequence of miR-1224-5p. Also, dual-luciferase reporter assay showed that luciferase activity was dramatically inhibited after co-transfection with miR-1224-5p and WT-FOXM1 3′UTR, whereas there was no significant change in the luciferase activity of MUT-FOXM1 3′UTR after miR-1224-5p overexpression (Fig. 5B, C). Additionally, FOXM1 expression was dramatically higher in CRC tissues than in normal colorectal tissues (Fig. 5D, E), as predicted by GEPIA and TCGA datasets. The mRNA and protein levels of FOXM1 were significantly upregulated in CRC tissues or HCT-116 and SW480 cells relative to paracancerous normal intestinal tissues or NCM460 cells (Fig. 5F, H, I). Meanwhile, it was also found that miR-1224-5p expression was negatively correlated with FOXM1 in CRC tissues (Fig. 5G). Furthermore, western blot results confirmed that FOXM1 protein expression was significantly decreased by miR-1224-5p mimic but increased by miR-1224-5p inhibitor (Fig. 5J). These evidences manifested that miR-1224-5p interacted with FOXM1 in CRC cells.

### MiR-1224-5p inhibited CRC progression and cell glycolysis via interacting with FOXM1

**Fig. 6:**
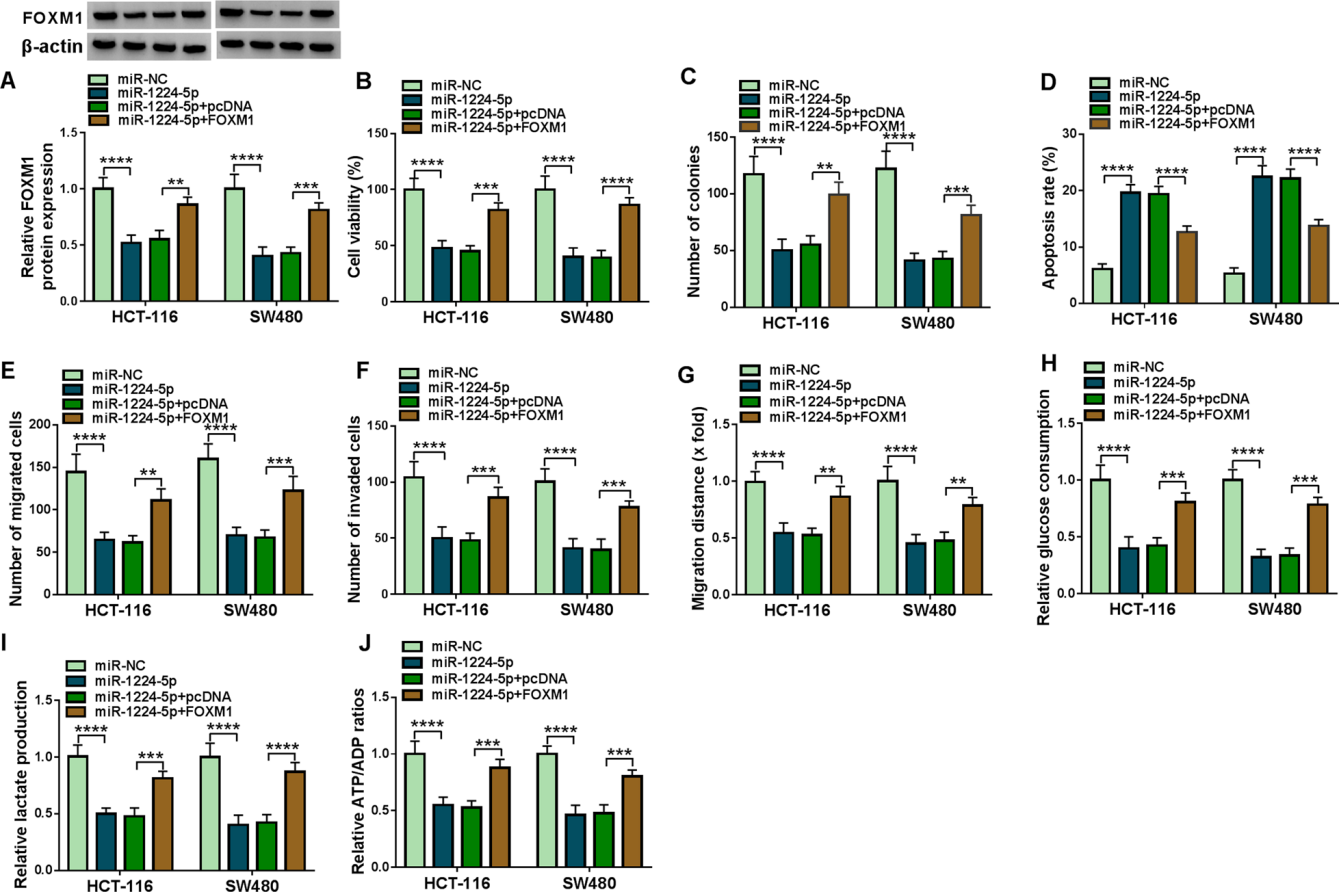
MiR-1224-5p mimic inhibited CRC cell processes by binding to FOXM1. **A** The impacts between miR-1224-5p mimic and FOXM1 overexpression on FOXM1 protein expression were disclosed by western blot. **B**, **C** The influences between miR-1224-5p and ectopic FOXM1 expression on the proliferation of HCT-116 and SW480 cells were revealed by MTT and cell colony formation assays. **D** Flow cytometry analysis was performed to analyze the impacts between miR-1224-5p mimic and FOXM1 overexpression on the apoptosis of HCT-116 and SW480 cells. **E**, **G** Transwell migration and wound-healing assays were carried out to determine the effects between miR-1224-5p mimic and FOXM1 overexpression on the migration of HCT-116 and SW480 cells. **F** The impacts between miR-1224-5p and FOXM1 overexpression on cell invasion were disclosed by transwell invasion assay in HCT-116 and SW480 cells. **H**–**J** The impacts between miR-1224-5p and FOXM1 overexpression on cell glycolysis were unveiled by glucose assay kit, lactate assay kit and ADP/ATP ratio assay kit in HCT-116 and SW480 cells. **P  < 0.01, ***P  < 0.001 and ****P  < 0.0001

Whether miR-1224-5p modulated CRC cell malignancy by binding to FOXM1 was further analyzed. Results initially showed that miR-1224-5p mimic downregulated FOXM1 protein expression, whereas FOXM1 overexpression reversed this impact (Fig. 6A). Subsequently, miR-1224-5p mimic repressed cell viability and colony-forming ability, but these effects were restored after FOXM1 overexpression (Fig. 6B, C). MiR-1224-5p mimic promoted cell apoptosis; however, ectopic FOXM1 expression attenuated this impact (Fig. 6D). The migration and invasion of HCT-116 and SW480 cells were also suppressed after transfection with miR-1224-5p mimic, but FOXM1 overexpression restrained these influences (Fig. 6E–G). Additionally, miR-1224-5p mimic repressed glucose uptake and lactate production, while enforced FOXM1 expression impaired these effects (Fig. 6H, I). ADP/ATP ratio kit assay showed that the value of ATP/ADP was reduced by miR-1224-5p mimic, whereas FOXM1 overexpression reversed this effect (Fig. 6J). These findings demonstrated that miR-1224-5p modulated CRC cell processes via associating with FOXM1.

### Circ-RNF121 silencing downregulated FOXM1 expression by binding to miR-1224-5p

**Fig. 7:**
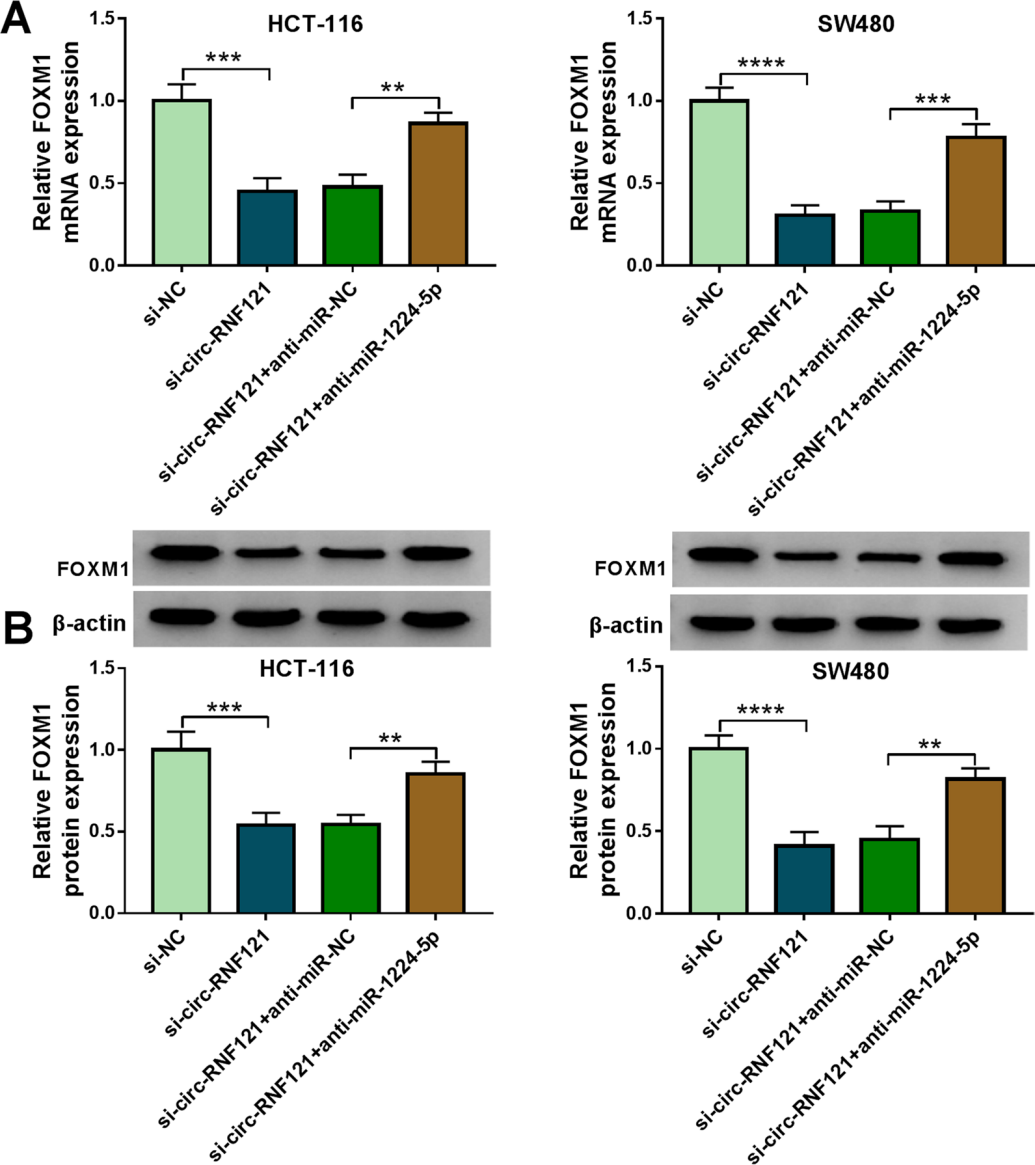
Circ-RNF121 regulated FOXM1 expression through binding to miR-1224-5p. **A**, **B** The effects between circ-RNF121 silencing and miR-1224-5p inhibitor on the mRNA and protein levels of FOXM1 were determined by qRT-PCR and western blot, respectively, in HCT-116 and SW480 cells. **P  < 0.01, ***P  < 0.001 and ****P  < 0.0001

Having proved the binding relationship of miR-1224-5p with circ-RNF121 or FOXM1, we further investigated whether circ-RNF121 modulated FOXM1 expression via sponging miR-1224-5p. Results showed that circ-RNF121 silencing decreased the mRNA and protein levels of FOXM1, while these effects were attenuated after miR-1224-5p knockdown (Fig. 7A, B), which suggested that circ-RNF121 modulated FOXM1 expression via sponging miR-1224-5p.

### Circ-RNF121 knockdown repressed tumor formation in vivo

**Fig. 8:**
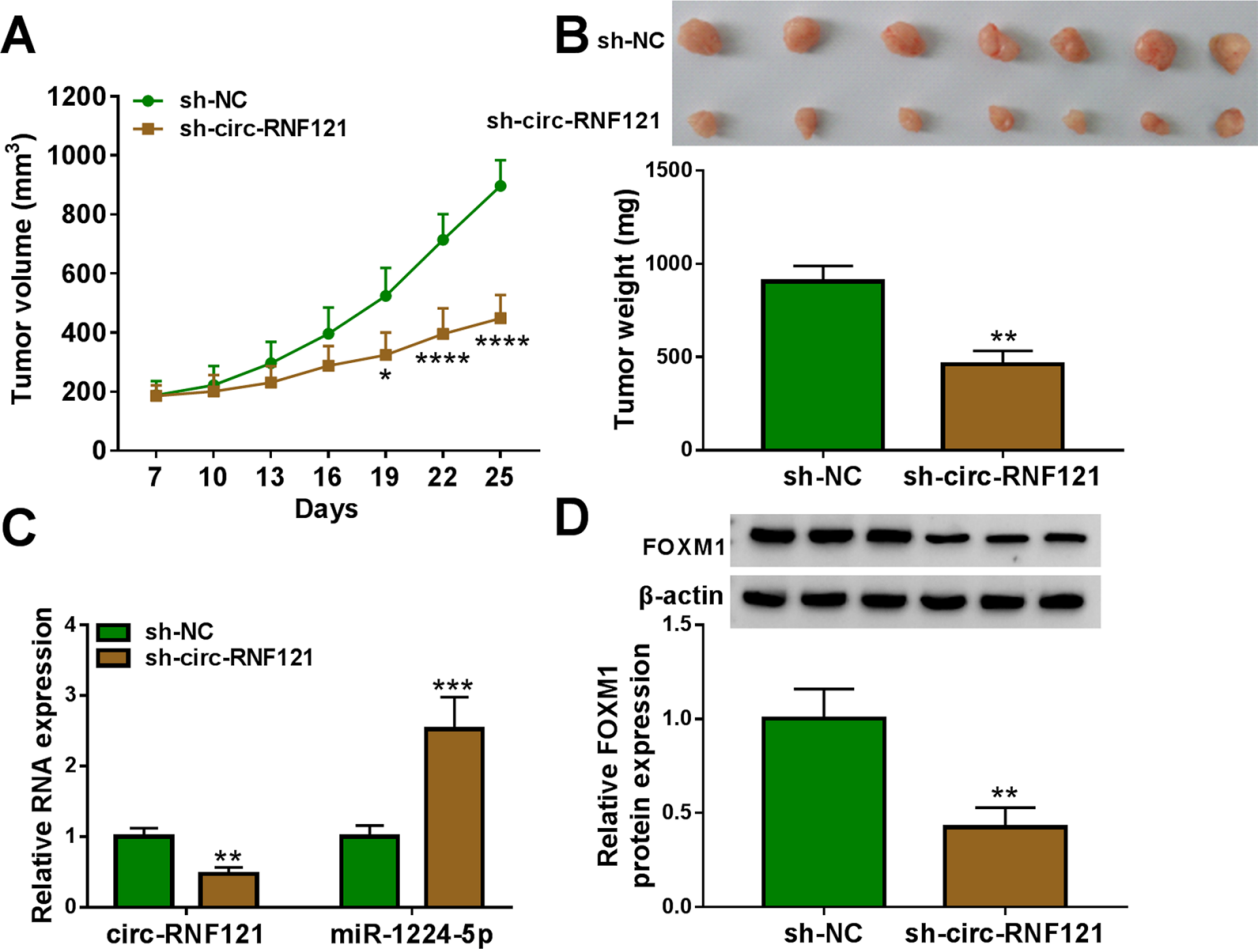
Circ-RNF121 silencing inhibited tumor growth in vivo. **A**, **B** The effects of circ-RNF121 silencing on the volume and weight of tumors in vivo were disclosed. **C** The impacts of circ-RNF121 silencing on the expression of circ-RNF121 and miR-1224-5p were determined by qRT-PCR in the forming tumors from sh-circ-RNF121 or sh-NC group. **D** The effect of circ-RNF121 knockdown on FOXM1 protein expression was analyzed by western blot in the primary tumors from SW480 cells. *P  < 0.05, **P  < 0.01, ***P  < 0.001 and ****P  < 0.0001

The effects of circ-RNF121 silencing on tumor growth in vivo were further disclosed. Results showed that tumor volume and weight were dramatically smaller or lighter in sh-circ-RNF121 transfection group than in sh-NC transfection group (Fig. 8A, B). Additionally, circ-RNF121 knockdown significantly downregulated circ-RNF121 expression (Fig. 8C), suggesting sh-circ-RNF121 was effective in knocking down circ-RNF121 expression in the forming tumors. The data from Fig. 8C, D showed that circ-RNF121 silencing notably upregulated miR-1224-5p expression and downregulated FOXM1 protein expression. Furthermore, we found that decreasing circ-RNF121 expression led to significant expression inhibition of HK2 and PKM2 in the forming tumor tissues (Additional file 2: Figure S2), two key glycolytic enzymes. These findings demonstrated that circ-RNF121 silencing inhibited tumor formation via regulating miR-1224-5p and FOXM1 expression in vivo.

### Circ-RNF121 was secreted through incorporating into exosomes in CRC cells

**Fig. 9:**
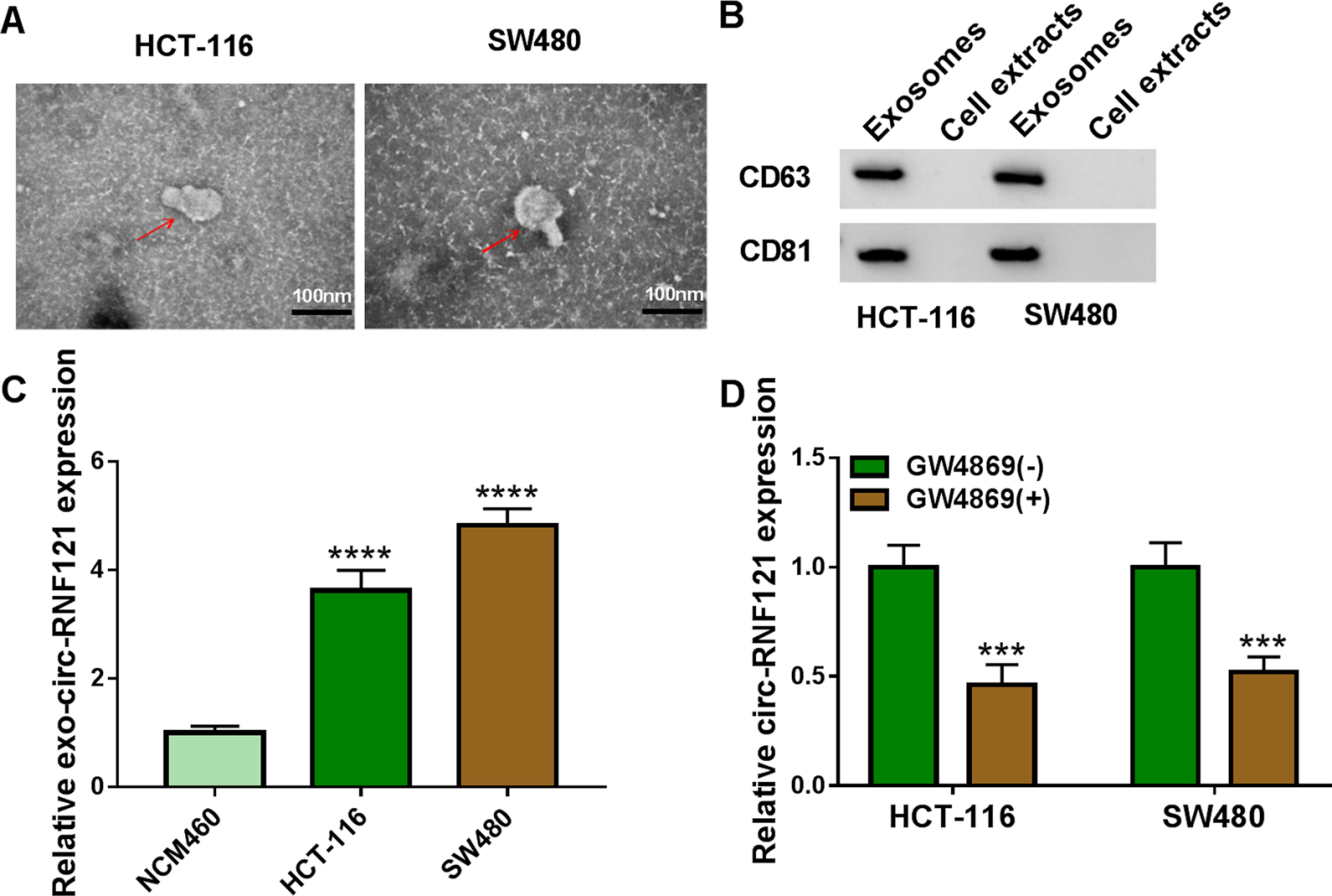
Extracellular circ-RNF121 was secreted by being packaged into exosomes in CRC cells. **A** TEM was performed to identify the structure of extracted vesicles. **B** Western blot was carried out to detect the expression levels of CD63 and CD81 in the extracted vesicles and cell extracts. **C** The expression of circ-RNF121 was determined by qRT-PCR in these exosomes from NCM460, HCT-116 and SW480 cells. **D** Circ-RNF121 expression in these exosomes isolated from HCT-116 and SW480 cells treated with GW4869(+) or GW4869(−) was determined by qRT-PCR. Exo-circ-RNF121: the circ-RNF121 derived from exosomes. GW4869(+): both HCT-116 and SW480 cells were treated with GW4869; GW4869(−): both HCT-116 and SW480 cells were not treated with GW4869. ***P  < 0.001 and ****P  < 0.0001

Exosomes were a kind of important mediators of intercellular communication. Whether circ-RNF121 was secreted via incorporating into exosomes was revealed in this part. Results firstly showed that extracted vesicles showed similar structure with exosomes (Fig. 9A), and exosomal protein makers (CD63 and CD81) were expressed in the isolated vesicles rather than in cell extracts (Fig. 9B), suggesting that exosomes from CRC cells were successfully isolated. Subsequently, circ-RNF121 expression was significantly upregulated in the exosomes from HCT-116 and SW480 cells compared with in these exosomes from NCM460 cells (Fig. 9C). On the contrary, the circRNA was obviously downregulated in the exosomes from HCT-116 and SW480 cells treated with GW4869 compared with control group (Fig. 9D). These findings suggested that circ-RNF121 was secreted by being packaged into exosomes in CRC cells.

## Discussion

CircRNA participates in the progression of CRC. For instance, circ_0026344 repressed CRC cell metastasis by sponging miR-183 [26]. Circ_101555 silencing suppressed CRC cell proliferation, while facilitated cell apoptosis via binding to miR-597-5p [27]. Additionally, Zhang et al. also explained circ_0007534 contributed to CRC cell metastasis and glucose metabolism by binding to miR-631 [28]. In this research, we found that circ-RNF121 knockdown restrained CRC progression and cell glucose metabolism via regulating miR-1224-5p/FOXM1 axis.

Previous researches have revealed the importance of circ-RNF121 in tumor development [8, 29]. For example, Yao et al. explained that circ_100876 was overexpressed in lung cancer tissues, and its upregulation was related to lymphatic metastasis [8]. Jin et al. showed that circ_100876 could inhibit the proliferation of osteosarcoma cancer cells via binding to miR-136 [9]. Additionally, circ_100876 restrained the proliferation and metastasis of breast cancer cells through interacting with miR-361-3p [10]. Zhang et al. also explained that circ-RNF121 overexpression promoted cell proliferative and metastatic ability, while hindered cell apoptosis in CRC [30]. In the present work, we found that circ-RNF121 was augmented in CRC specimens and cell lines. Loss-of-function experiments showed that circ-RNF121 silencing suppressed cell proliferation and metastasis, while induced cell apoptosis in CRC, suggesting the promoting effect of circ-RNF121 on CRC cell malignancy. In addition, our data explained that circ-RNF121 knockdown restrained tumor growth in vivo. Warburg effect, a hallmark of cancer process, is characterized via altering glycolysis, and energy in this effect is provided by these cells that convert glucose into lactate under aerobic environment for cancers [31]. In this study, we found circ-RNF121 silencing suppressed glucose uptake and lactate production, and reduced the value of ATP/ADP, implicating the promoting role of circ-RNF121 in glycolysis. Considering the function of exosomes as carriers, whether circ-RNF121 was secreted by incorporating into exosomes was explored in CRC cells in this study. Results showed that circ-RNF121 was secreted through being packaged into exosomes.

CircRNA frequently functioned as a sponge of miRNA to modulate cancer progression [32]. Thus, to unveil the mechanism of circ-RNF121 in CRC progression, we screened circ-RNF121-associated miRNA. Results showed that miR-1224-5p interacted with circ-RNF121. Li et al. have already indicated that miR-1224-5p expression was decreased in CRC patients, and its mimic repressed CRC cell metastasis [21]. Song et al. also showed miR-1224-5p suppressed cell proliferation, migration and invasion in CRC [33]. In this research, it was found that miR-1224-5p expression was significantly downregulated in CRC tissues and cells. MiR-1224-5p inhibitor attenuated circ-RNF121 silencing-mediated CRC cell malignancy, implying that miR-1224-5p inhibited cell proliferation and metastasis. Our findings were consistent with the above results. Additionally, miR-1224-5p inhibitor hindered circ-RNF121 absence-mediated cell apoptosis and glucose metabolism, which suggested that miR-1224-5p inhibited CRC cell glycolysis and promoted cell apoptosis. Meanwhile, our data explained that circ-RNF121 modulated CRC cell malignancy via binding to miR-1224-5p.

Considering that mRNA frequently regulated cancer evolution via interacting with mRNA [34], the mRNA targeted by miR-1224-5p was further explored. As a result, we found miR-1224-5p targeted FOXM1. Previous data have shown FOXM1 promotes CRC development by cooperating with miR-149 [35] and miR-320 [36]. In this paper, FOXM1 was increased in CRC tissues and cells. FOXM1 overexpression impaired the influences of miR-1224-5p mimic on CRC development and cell glycolysis, suggesting the promoting effect of FOXM1 in CRC cell processes. Previous researches have shown that FOXM1 acted as an oncogene in CRC progression by facilitating cell proliferation, migration, invasion and glycolysis, and repressing cell apoptosis [37–40]. These evidences supported our conclusion. Simultaneously, our data suggested that miR-1224-5p regulated CRC cell processes through interacting with FOXM1. Furthermore, circ-RNF121 could regulate FOXM1 expression through sponging miR-1224-5p.

In conclusion, circ-RNF121 silencing repressed cell proliferation, metastasis and glycolysis but promoted cell apoptosis in CRC. Circ-RNF121 modulated CRC malignant progression via sponging miR-1224-5p. Additionally, miR-1224-5p repressed CRC cell malignancy via targeting FOXM1. Circ-RNF121 repression also hindered tumor growth in vivo. Furthermore, circ-RNF121 was secreted by being packaged into exosomes in CRC cells. Collectively, circ-RNF121 regulated CRC cell malignancy via miR-1224-5p/FOXM1 axis. Our results provided a theoretical basis for developing circRNA-mediated therapy for CRC.

## Supplementary Information

**Additional file 1: Figure S1** The sequencing chromatograms of original and mutated sites in Fig. 3C, D and Fig. 5B, C.

**Additional file 2: Figure S2**. The effects of circ-RNF121 knockdown on the expression of HK2 and PKM2 were determined by western blot in the primary tissues from SW480 cells. **P<0.01 and ***P<0.001.

**Additional file 3: Table S1.** Relationship between circ-RNF121 expression and clinicopathologic features of colorectal cancer patients.

## Data Availability

Please contact the correspondence author for the data request.
