# Supplementary figures and images for: Circ-RNF121 regulates tumor progression and glucose metabolism by miR-1224-5p/FOXM1 axis in colorectal cancer
Source: Cancer Cell Int. 2021 Nov 6;21:596. doi: 10.1186/s12935-021-02290-3 (PMC8572430; doi:10.1186/s12935-021-02290-3)

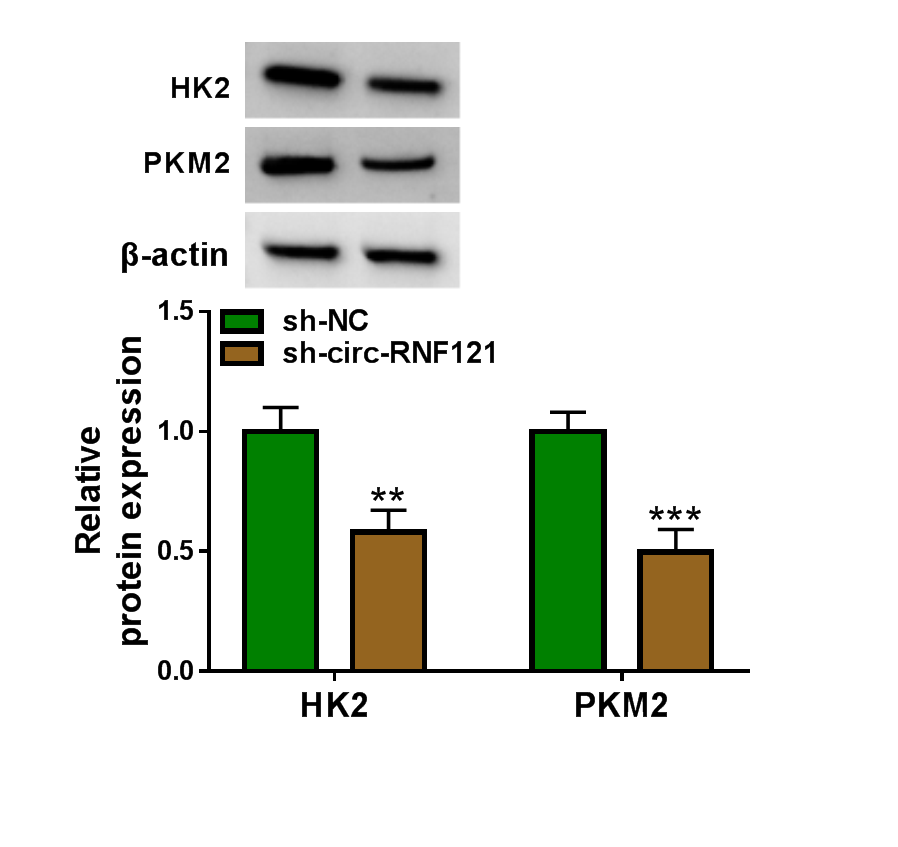

Supplement: Supplementary file 1 — Additional file 1: Figure S1 The sequencing chromatograms of original and mutated sites in Fig. 3C, D and Fig. 5B, C. [file 12935_2021_2290_MOESM1_ESM.tif]
