# Supplementary material for: Circ-RNF121 regulates tumor progression and glucose metabolism by miR-1224-5p/FOXM1 axis in colorectal cancer
Source: Cancer Cell Int. 2021 Nov 6;21:596. doi: 10.1186/s12935-021-02290-3 (PMC8572430; doi:10.1186/s12935-021-02290-3)
Supplement: Supplementary file 2 — Additional file 2: Figure S2. The effects of circ-RNF121 knockdown on the expression of HK2 and PKM2 were determined by western blot in the primary tissues from SW480 cells. **P<0.01 and ***P<0.001. [file 12935_2021_2290_MOESM2_ESM.pdf]

The original western blots of Fig5

G

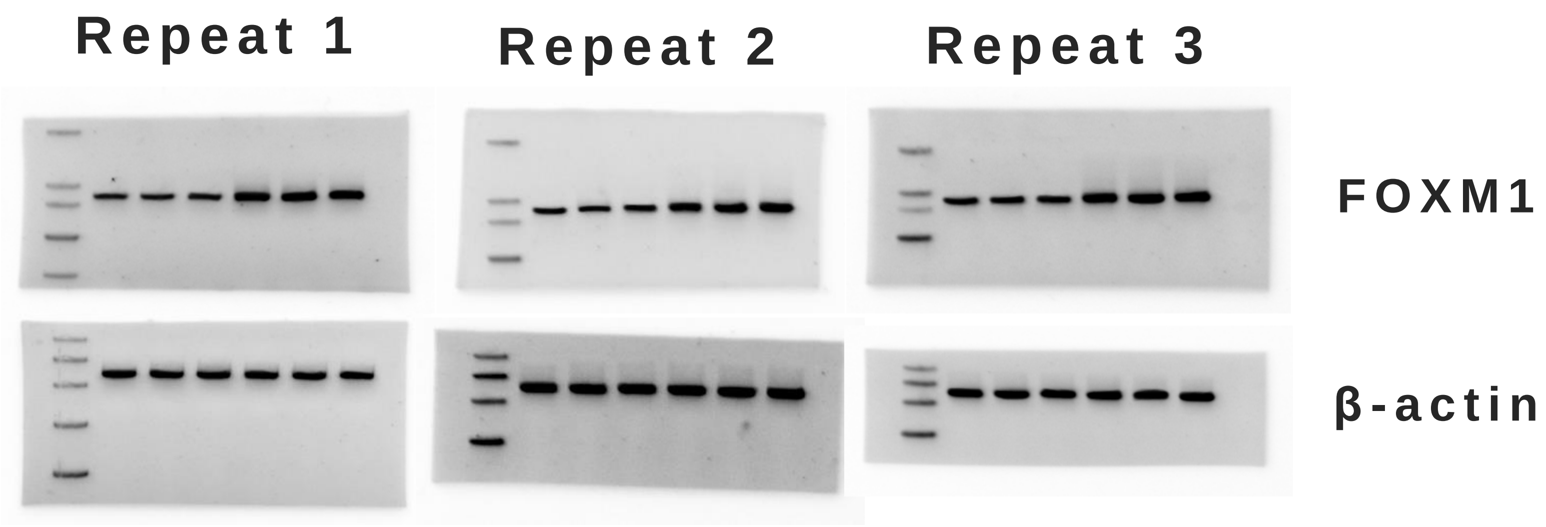

The original western blots of Fig5

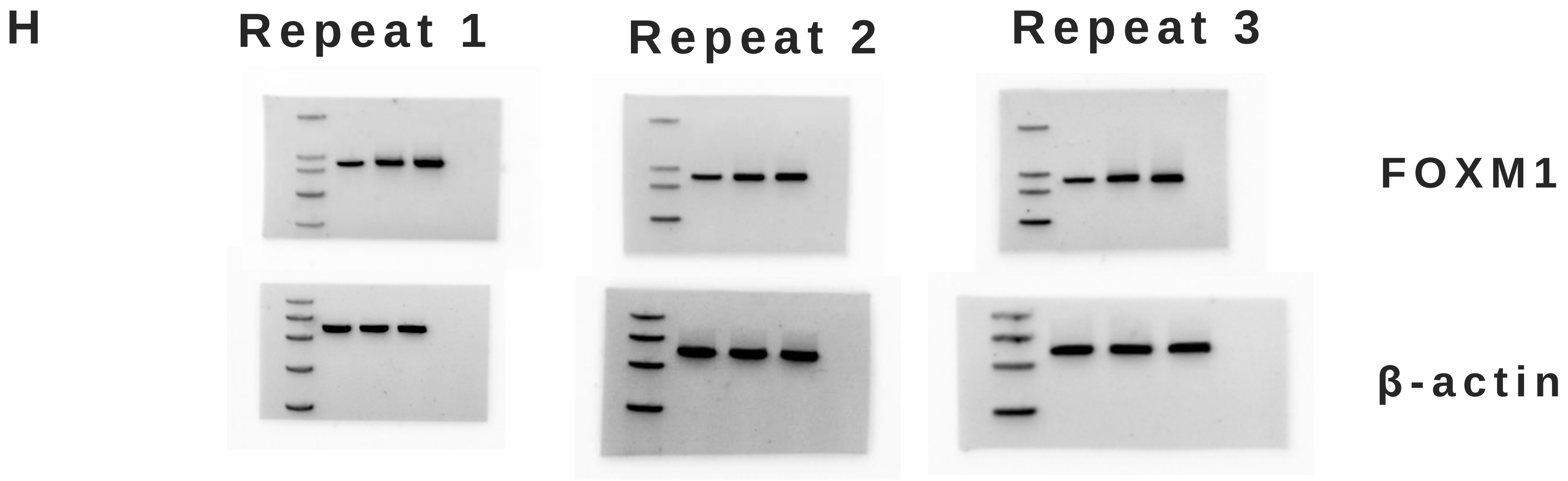

The original western blots of Fig5

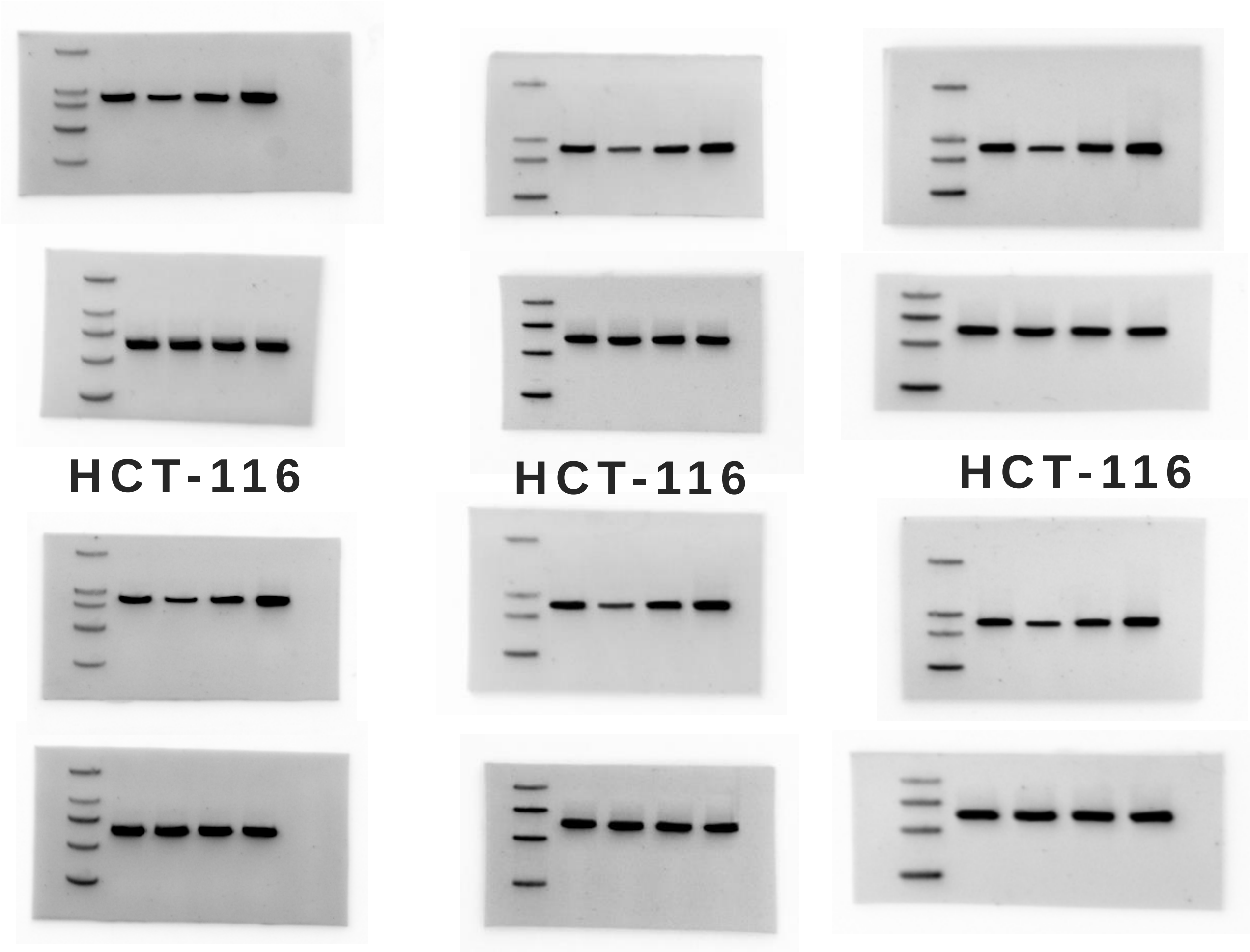

The original western blots of Fig6

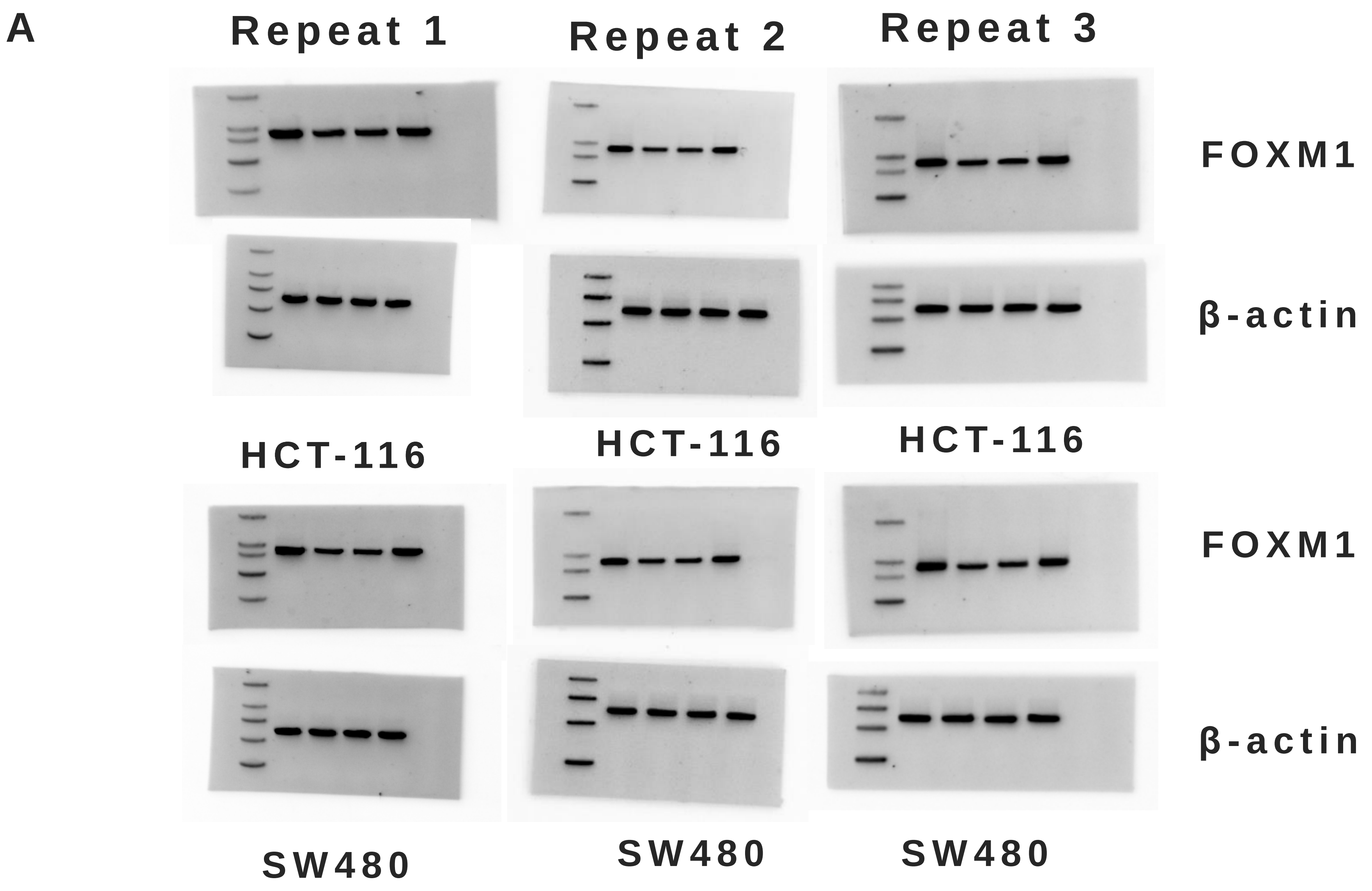

The original western blots of Fig7

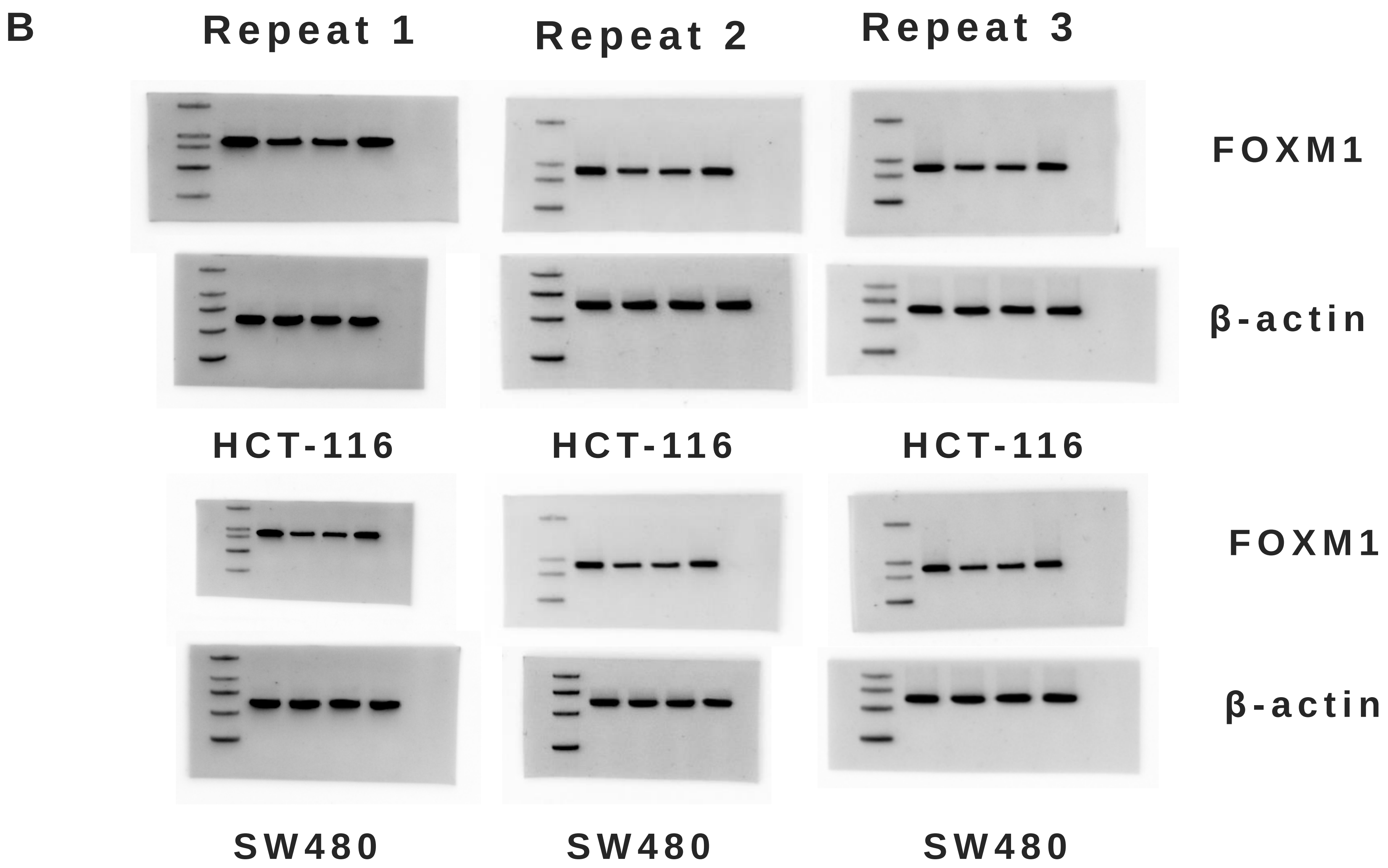

The original western blots of Fig8

D

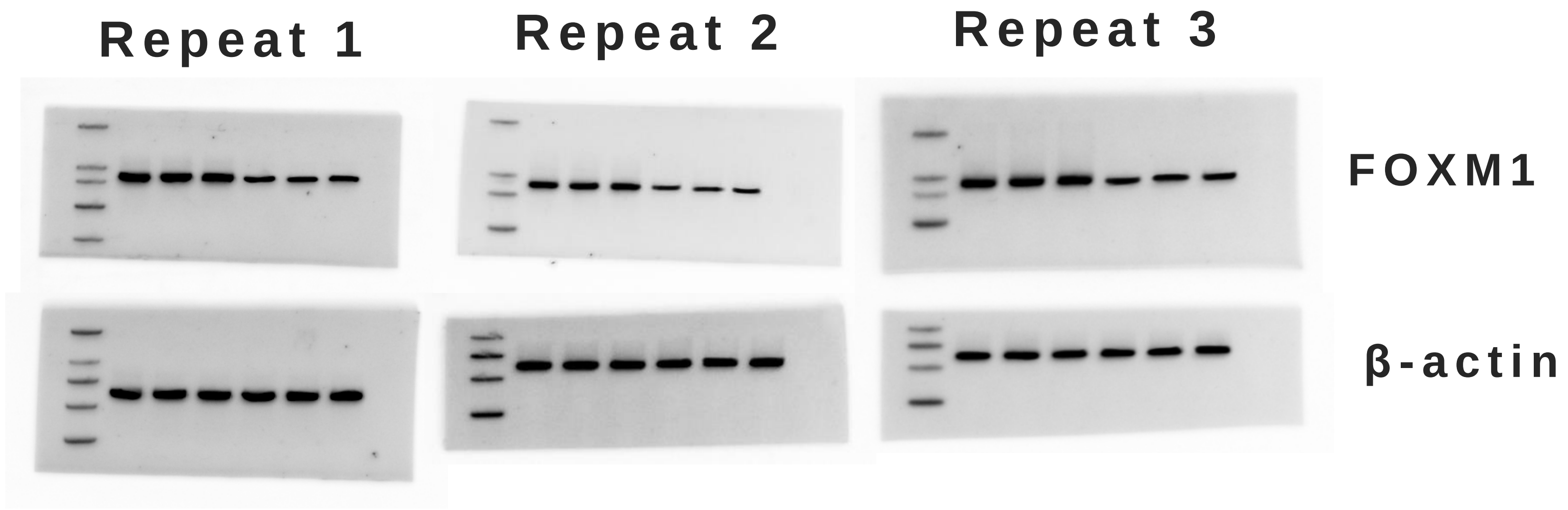

The original western blots of Fig9

B

Repeat 1 Repeat 2 Repeat 3

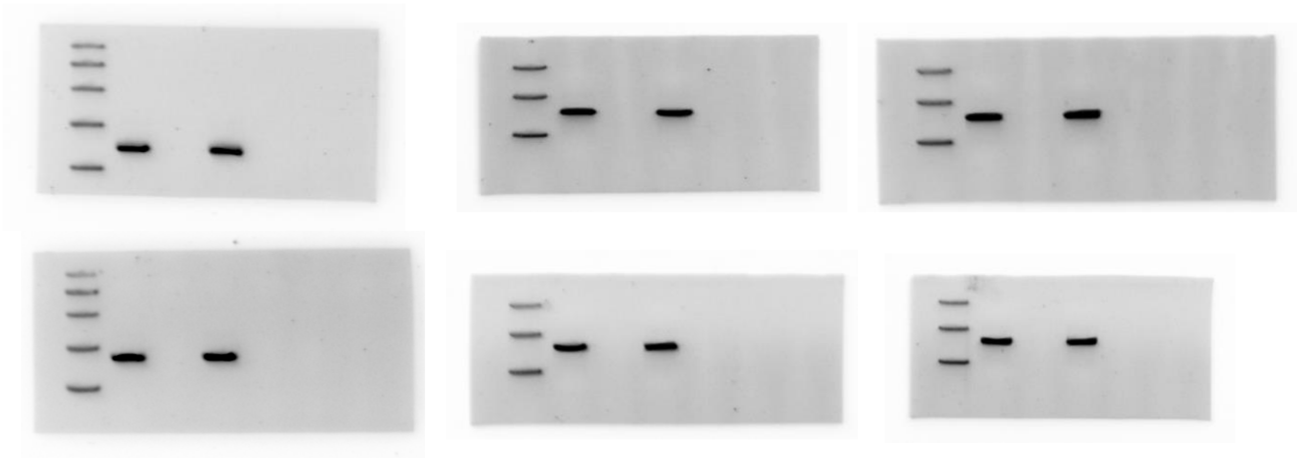

CD63

CD81
