# Supplementary material for: Circ-RNF121 regulates tumor progression and glucose metabolism by miR-1224-5p/FOXM1 axis in colorectal cancer
Source: Cancer Cell Int. 2021 Nov 6;21:596. doi: 10.1186/s12935-021-02290-3 (PMC8572430; doi:10.1186/s12935-021-02290-3)
Supplement: Supplementary file 3 — Additional file 3: Table S1. Relationship between circ-RNF121 expression and clinicopathologic features of colorectal cancer patients. [file 12935_2021_2290_MOESM3_ESM.doc]

Relationship between circ-RNF121 expression and clinicopathologic features of colorectal cancer patients

|  | Characteristics  n=29 | circ-RNF121 expression | | *P* value^a^ |
| --- | --- | --- | --- | --- |
|  |  | Low(n=14) | High(n=15) |  |
| Gender |  |  |  | 0.4661 |
| Female | 14 | 8 | 6 |  |
| Male | 15 | 6 | 9 |  |
| Age (years) |  |  |  | 0.4621 |
| ≤60 | 12 | 7 | 5 |  |
| >60 | 17 | 7 | 10 |  |
| TNM grade |  |  |  | 0.0092* |
| I+II | 13 | 10 | 3 |  |
| III+IV | 16 | 4 | 12 |  |
| Lymph node metastasis |  |  |  | 0.0078* |
| Positive | 18 | 5 | 13 |  |
| Negative | 11 | 9 | 2 |  |
| Tumor size |  |  |  | 0.0209* |
| ≤3 cm | 10 | 8 | 2 |  |
| >3 cm | 19 | 6 | 13 |  |

TNM, tumor-node-metas-tasis; **P* < 0.05 ^a^Chi-square test
